# IPC prediction of patent documents using neural network with attention for hierarchical structure

**DOI:** 10.1371/journal.pone.0282361

**Published:** 2023-03-02

**Authors:** Yuki Hoshino, Yoshimasa Utsumi, Yoshiro Matsuda, Yoshitoshi Tanaka, Kazuhide Nakata

**Affiliations:** 1 Tokyo Institute of Technology, Meguro City, Tokyo, Japan; 2 Rakuten Group, Inc., Setagaya City, Tokyo, Japan; Taipei Medical University, TAIWAN

## Abstract

International patent classifications (IPCs) are assigned to patent documents; however, since the procedure for assigning classifications is manually done by the patent examiner, it takes a lot of time and effort to select some IPCs from about 70,000 IPCs. Hence, some research has been conducted on patent classification with machine learning. However, patent documents are very voluminous, and learning with all the claims (the part describing the content of the patent) as input would run out of the necessary memory, even if the batch size is set to a very small size. Therefore, most of the existing methods learn by excluding some information, such as using only the first claim as input. In this study, we propose a model that considers the contents of all claims by extracting important information for input. In addition, we focus on the hierarchical structure of the IPC, and propose a new decoder architecture to consider it. Finally, we conducted an experiment using actual patent data to verify the accuracy of the prediction. The results showed a significant improvement in accuracy compared to existing methods, and the actual applicability of the method was also discussed.

## 1 Introduction

Improving the efficiency of patent examination is a very important issue. Currently, nearly three million patent applications are filed worldwide every year. All of this is manually reviewed by patent examiners, which requires a lot of work. This patent examination process takes a very long time and varies from country to country; in many regions, it takes more than six months and, in some countries, it takes more than a year. During the examination period, the right to a patent is not granted, and the efficiency of the examination is considered important.

Each patent is assigned several international patent classification (IPC) according to its field. During examination and application, the IPC is used to search for similar patents and existing technologies. It is essential to grant the IPC correctly because an incorrectly granted IPC can result in a patent being granted incorrectly. Creating IPC prediction models using machine learning will improve the efficiency of patent examination. There are three reasons for that. First, patent examiners manually assign IPCs, so automating or semi-automating IPC prediction to assist the examiners will lead to more efficient examinations. In addition, because the predicted IPC indicates the field of the patent to be examined, it is possible to assign the examination to a patent examiner who is familiar with the field at the beginning of the examination. Finally, because it is useful for searching for similar patents necessary for examination, it may lead to the efficiency of the patent examination itself.

IPC prediction can be viewed as a multi-label classification problem for natural language possessing a hierarchical structure. First of all, although the specific structure of IPC will be introduced in the next section, IPC has a hierarchical structure. And, multiple IPCs are assigned to a single patent. So, IPC prediction model actually predicts multi-label classification with a hierarchical structure. Each region also provides its own classification expressions, such as cooperative patent classification (CPC) and file index (FI), and they have also a hierarchical structure. Therefore, this prediction model can also be used to predict CPC or FI, and so on.

In recent years, machine learning such as deep learning has been applied to various fields, especially attention-based methods have increased in recent years [1, 2]. There are several studies on IPC prediction, but most of them use basic machine learning methods [3–5]. In recent studies, some methods use deep learning method [6, 7]. More generally, multi-label text classification using hierarchical structures is also performed using deep learning models [8–10]. However, these methods do not address the problem of long sentences, which is a challenge unique to patents.

The contributions of this study are as follows.

1. Point out problems with predictions using the first claim and proposing a method for extracting the necessary information from the full claims.
2. Propose a decoder that uses the hierarchical structure of IPC.
3. Verify the availability of the model using actual patent data.

The structure of this paper is as follows. First, in Section 2, we introduce the patent documents including IPC, etc. Section 3 introduces existing methods related to IPC prediction of patents. In Section 4, we introduce our proposed method based on Section 3, and in Section 5, we verify the effectiveness of the proposed method through experiments using real data. Finally, a summary of this study and future issues are presented.

## 2 Basic information on patents

First, we will describe the structure of a patent document. Patent documents have a lot of information such as “Inventors”, “Patent Numbers” and “IPC”. There are also texts about the content of the invention, which are expressed at various levels of granularity. For example, the “abstract” part contains a brief description of the invention in broad strokes. The “claims” part refers to the technical contents more specifically. From here, we will introduce these in turn.

The “abstract” section describes the general contents of the patent. There is no specification of how to write “abstracts”, and they range from detailed to very simple. For example, the abstract section of a certain patent is as follows.

Formulations of anti-VLA-1 antibodies are described.

It is not possible to infer from this statement what kind of technology this is. Therefore, the “abstract” part is not sufficient to understand the patent.

The “claims” is the part that shows the essential contents of the patent and is characterized by the fact that it consists of multiple claims as follows.

1. A compound of Formula (I) [Chemical] or a stereochemically isomeric form thereof, wherein R is hydrogen or fluoro, or an addition salt thereof.
2. The compound according to claim 1 wherein R is fluoro and the compound is a racemic mixture, or an addition salt thereof.
3. A pharmaceutical composition comprising a therapeutically effective amount of a compound as defined in claim 1 and a pharmaceutically acceptable carrier.
4. A compound as defined in claim 1 for use in the treatment of pulmonary arterial hypertension, pulmonary fibrosis, or irritable bowel syndrome.

This example is a relatively short sentence with a small number of claims, and there are some long sentences with more than 100 claims. Claims can be divided into two main patterns. The first is a claim that is complete by itself and is called an independent claim. In general, the first claim is always an independent claim, which gives you a general idea of the patent. The second is called a dependent claim, which is given in the form of a supplement to the preceding claims, as in the second and following claims above. Although a claim is a dependent claim, the claim may describe areas not previously mentioned. For example, the use as a drug is mentioned for the first time in the third claim given as an example. We have to read all claims to know what field of the invention is.

We also introduce the characteristics of IPC. IPC is generally described as consisting of four hierarchies. The hierarchy is referred to as sections, classes, subclasses, and groups, in order from the top. For example, in “H01F 1/01” H is a section called ELECTRICITY. Next, 01 is the class, and F is the subclass. Finally, “1/01” is a group, but there is actually a hierarchical structure here as well, with the main group before the “/” and the subgroups after. When subgroups are considered, the classification is broken down into the detailed category of “Magnets or magnetic bodies characterised by the magnetic materials therefore of inorganic materials” In this paper, all these classifications from sections to subgroups are collectively expressed as labels. All of these labels belong to just one of the hierarchies above. Although there are only eight types of sections, the number of such labels increases as one moves down the hierarchy, and because there are approximately 70,000 subclasses, prediction is more difficult toward the end of the hierarchy.

It is also important to note that multiple labels can be assigned. The IPC is assigned to inventions from a variety of perspectives such as material, manufacturing method, and usage. Inventions are often achieved by combining technologies from multiple fields. In 2013, the average number of IPCs granted per patent was approximately 4.16 labels, and some patents were granted more than 100 IPCs.

## 3 Related works

In the previous section, predicting IPC of patent documents can be viewed as a hierarchical multi-label classification problem because the task is to assign multiple labels with a hierarchical structure. In this section, we introduce existing methods for the hierarchical multi-label classification problem. Next, we introduce HARNN [10], which is used for multi-label text classification problems in general. Finally, we explain Patent BERT [7], which is an existing study on IPC assignment of patent documents.

### 3.1 Hierarchical multi-label text classification

There are some methods for hierarchical multi-label classification. One of them is a method using an SVM [11]. In this method, the loss function of SVM is changed according to the hierarchy, and the more distant the label, the larger the loss. When we use this method for multi-label classification problems, we must solve the binary classification problem for the number of labels. Since the number of IPCs is large, it will take a huge time to predict IPC with this method. Another method using decision trees was proposed [12]. In this paper, three decision tree methods are proposed, and each is evaluated. All of these methods are only used for solving general classification problems and are not suitable for text information. As text information is ordered and variable in length, it is very difficult to use these basic methods.

Several studies have used neural networks [8, 9, 13]. These methods have specific loss function [8] or allow variable length output in the manner of rnn [9]. Among them, the method with the highest prediction accuracy at present is called HARNN [10]. The encoder of this method is a bidirectional LSTM, and the decoder is a model that introduces a hierarchical structure. Decoders are roughly divided into two types: global and local. The global decoder predicts the labels of all levels at once, while the local decoder predicts the child levels of each level based on the predictions of the parent level. HARNN is currently state-of-the-art in the task of predicting labels for all hierarchies and is a model with very high prediction accuracy. Recentry, Transformer [14] are commonly used in natural language processing. However there are no research with Transformer based method which predict label with hierarchcal structure.

### 3.2 Patent labeling

Many studies have been done to predict IPC for patent documents. First, basic methods such as SVM and k-NN were used to classify patent documents [3]. Classification using Word2Vec was also studied [5]. It is also shown that other methods including neural networks have the potential to be adapted [4]. Deep learning was utilized for the first time in 2018, where the prediction accuracy of classification was confirmed using a CNN-based deep learning model [6].

Patent BERT [7] is a model based on BERT [15], which is widely used as a natural language processing model. BERT is a model that has been pre-trained using a large corpus for the transformer, and a fine-tuned version of BERT is now state-of-the-art in many fields. The decoder part needs to be created separately depending on the task, for BERT is a pre-trained model for the encoder only. Patent BERT is very simple, with only one layer of matrix calculation and sigmoid function. This model supports multi-labeling but does not consider the hierarchical structure at all.

## 4 Proposed method

**Fig 1:**
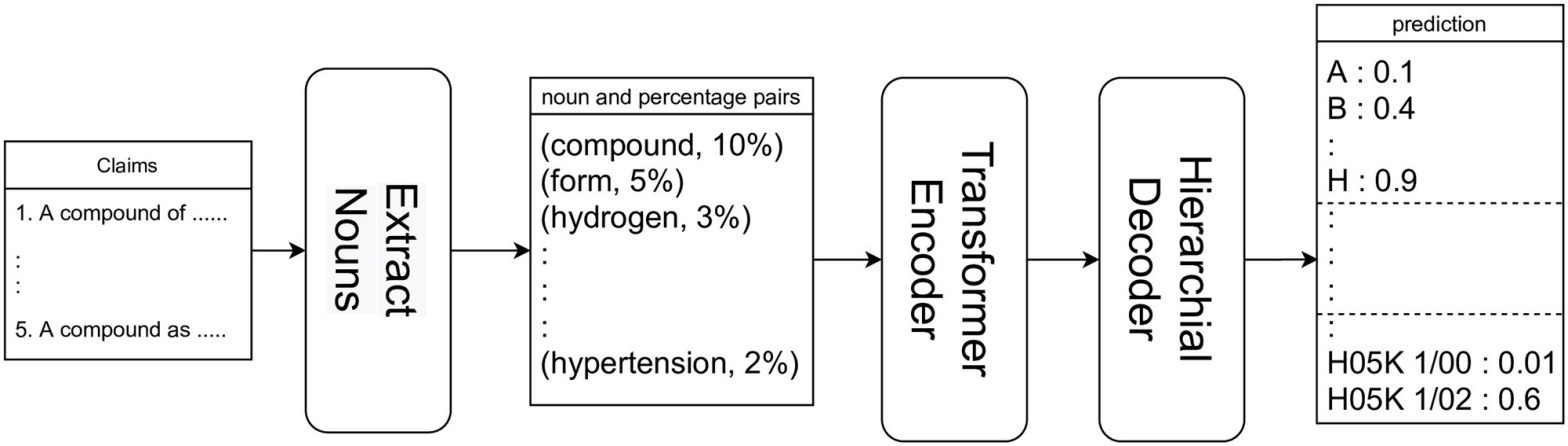
Overview of the proposed model.

In this section, we explain the specific method of the proposed patent document classification model. An outline of the proposed classification method is shown in Fig 1. First, nouns and their percentages are extracted from a large amount of textual information contained in the claims of patent documents. Second, the encoder generates the features of the target patent, and finally, the decoder performs the IPC prediction according to the features created by the encoder. In the following sections, we explain noun extraction in 4.1, the encoder in 4.2, and the decoder in 4.3.

### 4.1 Noun extraction

First, as mentioned earlier, the claims of patent documents are very long and sometimes many. In fact, the average number of tokens for the full text of the patent claims was very long (approximately 1200), and some of them were over 10,000 tokens. So if you try to input the full text consisting of 10,000 tokens, you will run out of RAM and will not be able to do so even if the batch size set to 1. When we look at the claims, we can hypothesize that the words specific to each field are mostly nouns, and in many cases, it is possible to predict IPC from nouns only. So, In the proposed method, nouns are extracted from all claims using morphological analysis.

To extract nouns, we use morphological analysis. Morphological analysis is the process of decomposing a sentence into words and their parts of speech. Morphological analysis has been studied in many languages, and at the same time, many morphological analysis tools have been released. Parts of speech vary from language to language, but in general, there are nouns, verbs, adjectives, etc., each of which plays a similar role in the language. Therefore, extracting nouns can produce similar results in any language and is expected to be effective in a variety of languages.

When only nouns are extracted, a sequence of nouns is obtained. Although the number of occurrences may have a significant impact to determine the patent content, the order of the nouns may be of little significance in determining the patent content. The number of occurrences is considered to be significantly affected by sentence length. For example, the meaning of ten nouns out of a thousand nouns would be different from that of ten nouns out of twenty nouns. Therefore, we calculated the percentage of occurrences by dividing it by the total number of noun occurrences in the actual input. We did not set stopwords and used all extracted nouns, for we considered that the general wording also differs by field. As a result, we were able to keep the average number of tokens to approximately 140, and because the memory requirement during training increases in the order of the square of the number of tokens in the model of the proposed method, we were able to reduce the memory requirement to approximately 1.4%.

### 4.2 Encoder

In this section, we explain the central computational structures of our model, attention, and transformer, in order. Next, we describe the specific encoder model that extracts the information about nouns and their proportions using the proposed method.

#### 4.2.1 Attention

The attention mechanism [16] is a general term for a method that calculates the importance of variable-length input by inner products or compresses input by importance, as shown in the following equation.

$$\alpha (q,K)=\text{Softmax}(qK^{T})$$ (1)

Attention(q,K,V)=α(q,K)V (2)

Here, *q*, *K*, *V* are called query, key, and value, respectively, and key and value often contain the same value. In Eq 1, we first take the inner product of *q* and each column vector of *K*. The closer the directions of the two vectors are, the larger the value of the inner product, so the value of the inner product can be regarded as a measure of the closeness or relevance of the elements. By applying the softmax function to the elements, we put them in the (0, 1) interval and normalize the sum to 1 so that it can be regarded as a proportion. Therefore, ***α***(*q*, *K*) can be regarded as a decomposition of q and K according to their degree of association. In the second equation, we take the product of ***α***(*q*, *K*) and V. Since *α* can be regarded as a proportion, we can regard it as taking a weighted average of V. Therefore, the above attention can be interpreted as a method of compressing values considering the relevance from the viewpoint of the query.

**Fig 2:**
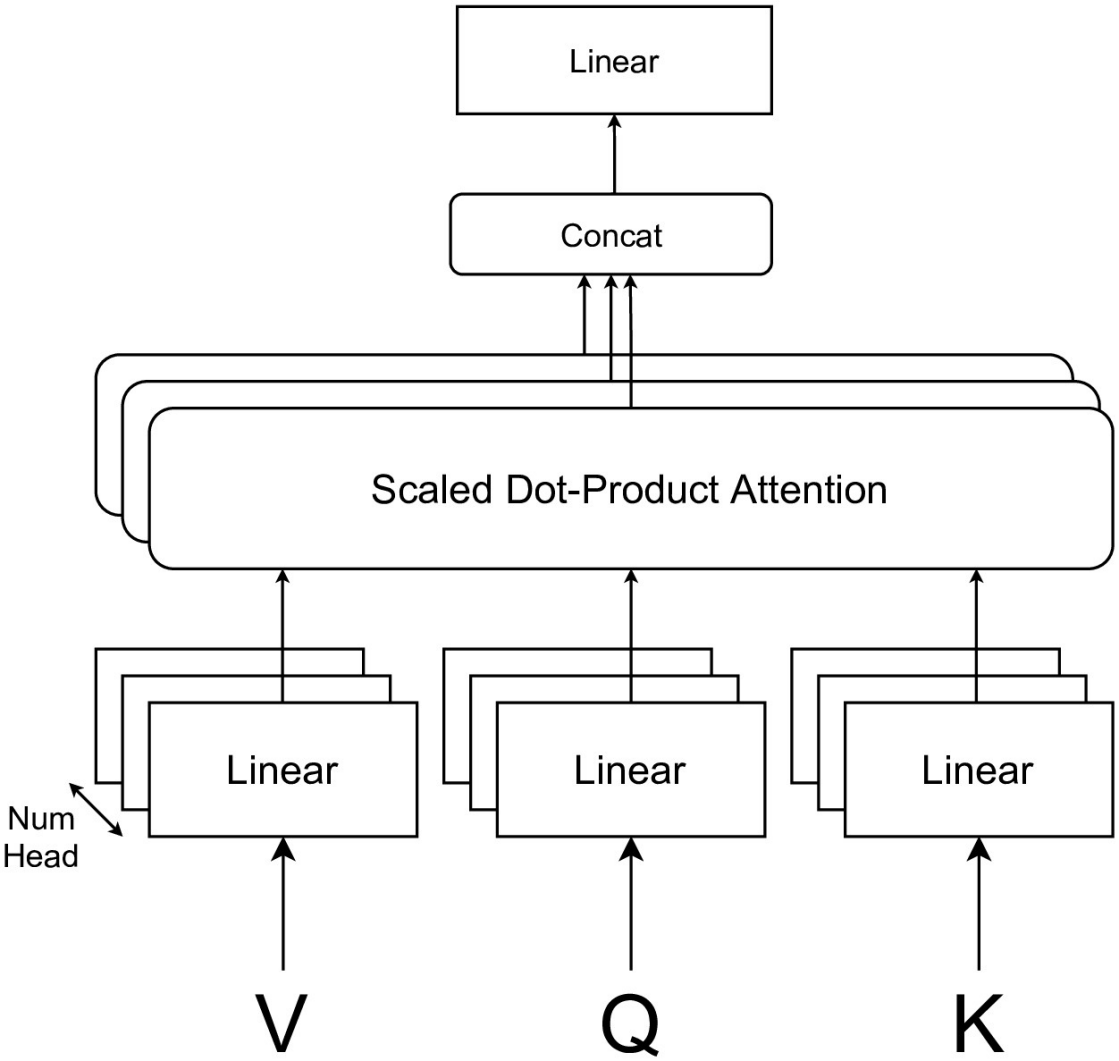
Structure of multi head attention.

Multi-head attention [14], a method that applies the attention mechanism and is widely used in the field of deep learning, especially in natural language processing, is shown in Fig 2. Scaled dot-product attention (SDPA) is represented as in Eq 3, and compared to the attention mechanism described above, there are two changes. First, the query *q* is matrixed, compressing the information from multiple perspectives and producing each result at once. Second, scaling by dividing by $\sqrt{d_{k}}$ encourages the output of size independent of the number of viewpoints.

$$\begin{array}{c} \text{SDPA}\left(Q,K,V\right)=\text{softmax}\left(\frac{QK^{T}}{\sqrt{d_{k}}}\right)V \end{array}$$ (3)

Multi-head attention (MHA), as shown in Fig 2, measures the relevance of various perspectives by performing Scaled Dot-Product Attention multiple times on the input in small dimensions, and compresses the information using different compression rates, thus The range of expression is expanding. In particular, self-dot attention, which uses the same value for all *Q*, *K*, *V*, is very powerful and is used not only in natural language processing but also in image recognition because it can be calculated for inputs of arbitrary length and can measure the relevance of inputs to each other. It requires considerable computation time and memory because it has to calculate the square of the length of the input.

The transformer is an iterative method that uses self-dot attention and simple dense layers introduced in the previous section. The unit structure is expressed as in Eq (4), where the normalization is expressed as Norm.

Trans(Q,K,V)=Norm(Q+relu(WNorm(MHA(Q,K,V)))) (4)

When used in natural language processing, the input words are converted into word vectors in the embedding layer and then converted into a sequence of vectors. In addition, relative (or absolute) positional information was added to each word using positional encoding. Then, by repeatedly using the unit structure of the transformer, it is possible to learn all inputs, including their relationships.

Set transformer [17] is the transformer minus positional encoding. They also suggest a method to compressing variable length input to a fixed length by using fixed length (often 1, in particular) parameters for the query. This model was shown to be effective for several tasks in the original study.

#### 4.2.2 Proposed encoder

Since the input of the model is a set of nouns and their percentages, the encoder is based on the set transformer. However, it is necessary to embed the percentage information because the percentage of nouns is considered as input here. We propose a percentage embedding method in the encoder as follows.

**Fig 3:**
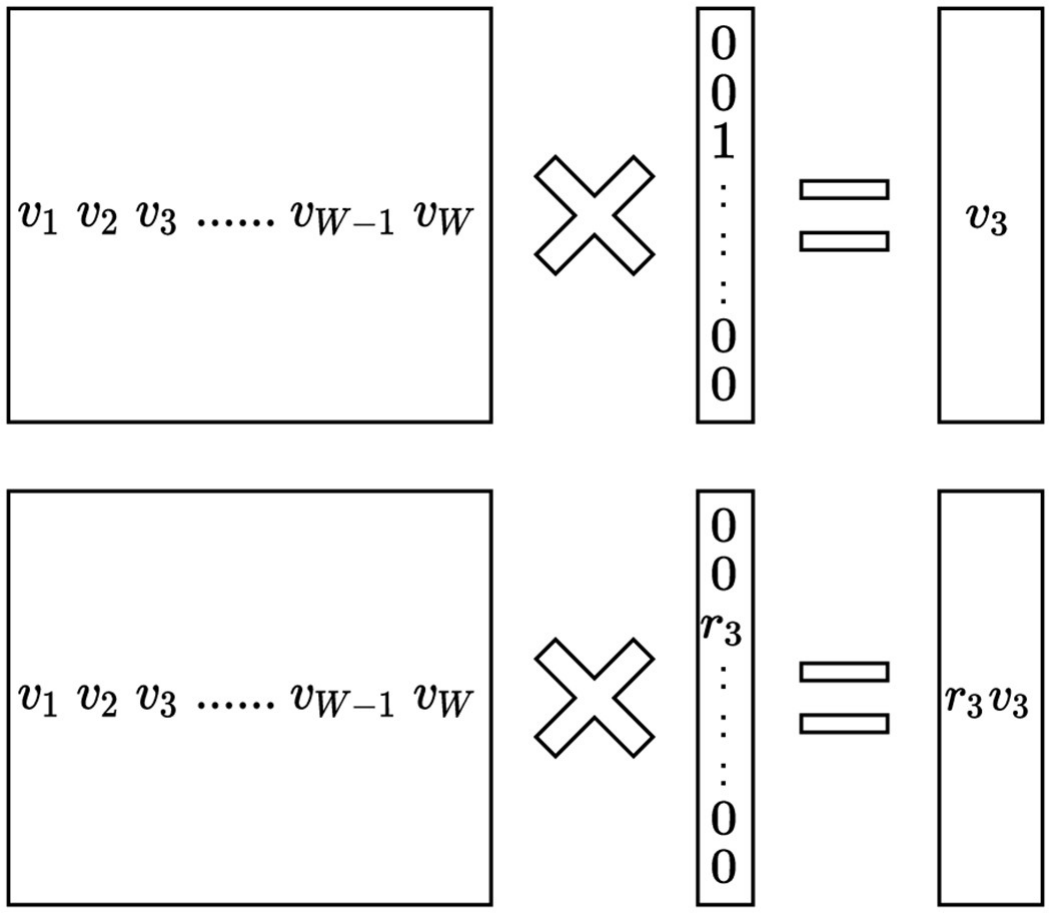
Interpretation of ratio encoding.

Word embedding can be regarded as a matrix calculation of the one-hot encoding vector and the matrix of the embedding vector, as shown in the upper part of Fig 3. If we extend this to consider the percentage information, we can convert the original one-hot encoding into a percentage, as shown in the bottom row. The output of the matrix calculation is the embedding vector multiplied by the percentage. Therefore, the calculation of the percentage encoding layer is *r*_*i*_***e***_***i***_, where ***e***_***i***_ is the embedding vector of the *i*th element, and *r*_*i*_ is the percentage.

**Fig 4:**
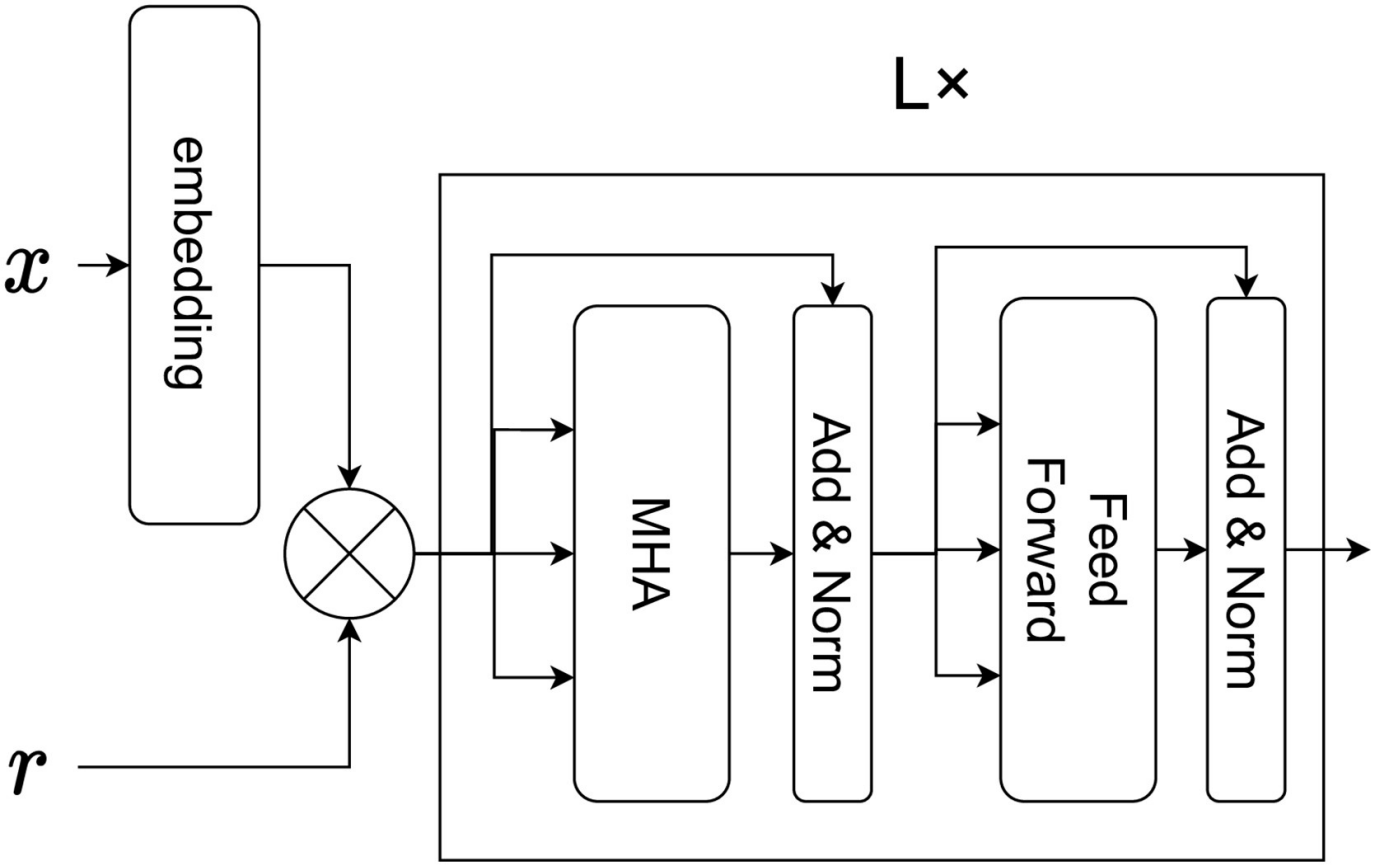
Structure of encoder.

Based on the above, the overall picture of the encoder is as follows. The forward propagation calculation of the encoder including percentage encoding and transformer is as shown in Eqs (5)–(7). The figure can be represented as Fig 4, where the meaning of the word and its percentage can be considered by first converting the word to an embedding vector and then multiplying by the percentage. The words are input to the transformer encoder, which includes an attention mechanism so that it can learn the interaction between the words. For example, to assign IPC, which means manufacturing of hats, it is considered meaningful that the two words “hat” and “manufacturing” are together. In such a case, it is necessary to learn the relationship between the two words, and the attention mechanism of the transformer plays an important role in learning the relationship.

$$e_{i}=\text{Embedd}(x_{i})$$ (5)

$$h_{i}^{0}=r_{i}e_{i}$$ (6)

$$H_{i+1}=\text{Trans}(H_{i},H_{i},H_{i})$$ (7)

### 4.3 Decoder

We propose a decoder to utilize hierarchical information. It is assumed that the IPC is very different for each field. For example, in the field of chemistry, the names of chemicals may be considered, and in the field of mechanics, it may be important to know what the purpose is. Therefore, it is expected that IPC prediction should be processed in a way that is specific to each field, and a decoder with a hierarchical structure is proposed according to the IPC to be targeted.

**Fig 5:**
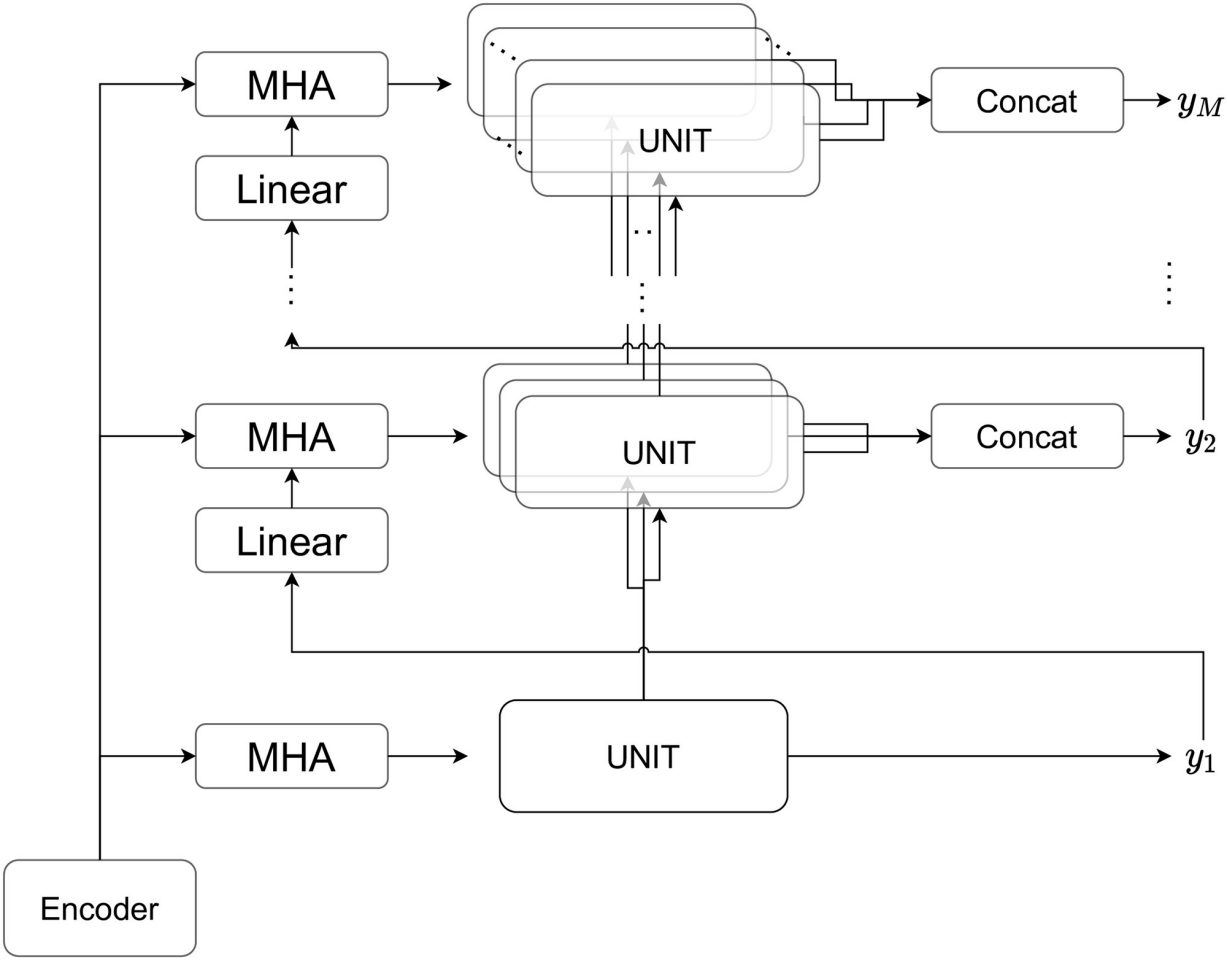
Structure of decoder.

The outline of the proposed decoder is shown in Fig 5. The UNIT part of Fig 5 is the unit process of the decoder, where each unit predicts whether or not each child label has been assigned. First, the shallowest layer performs the same processing and predicts the section. In the second level, there are as many units as the number of sections, and the class within each section is predicted. At the third level, subclasses within each class are predicted, and so on. Hence, each node performs a unique process within each hierarchy, and the computation can be adapted to each field.

**Fig 6:**
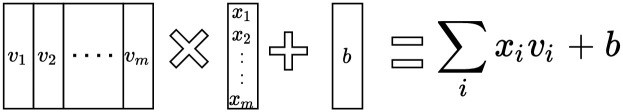
Image to make a query.

I will explain Attention. Attention was originally thought to be the most expressive method to be implemented in each unit. The advantage of attention is that it can be computed using all the outputs of the encoder without compression, but this requires memory and computation time. Here, the number of units is as many as the number of nodes of IPCs to be predicted, and even if the number of IPCs to be predicted is limited to approximately one thousand, it must be performed several thousand times. Therefore, the attention mechanism is considered to be global because the GPU memory would be insufficient if it is implemented in all units. Since attention is a compression method that calculates the importance according to the query, the query is important. In this case, the query is a linear transformation of the output of the previous level by a trainable matrix and vector. As shown in Fig 6, the column vectors of the matrices in the linear transformation can be regarded as vectors corresponding to each prediction target in the previous hierarchy and are considered to be weighted sums of the outputs of the parent nodes. In other words, the larger the prediction result of the parent node, the more it reflects the vector. As the output of the parent node is obtained by the sigmoid function (not softmax function), the query tends to have a large value when it is predicted that many labels will be given in the previous level. This is in line with the intuition that the more labels a node has, the more information it has. This makes it possible to take global attention into account the predicted values of the previous level.

**Fig 7:**
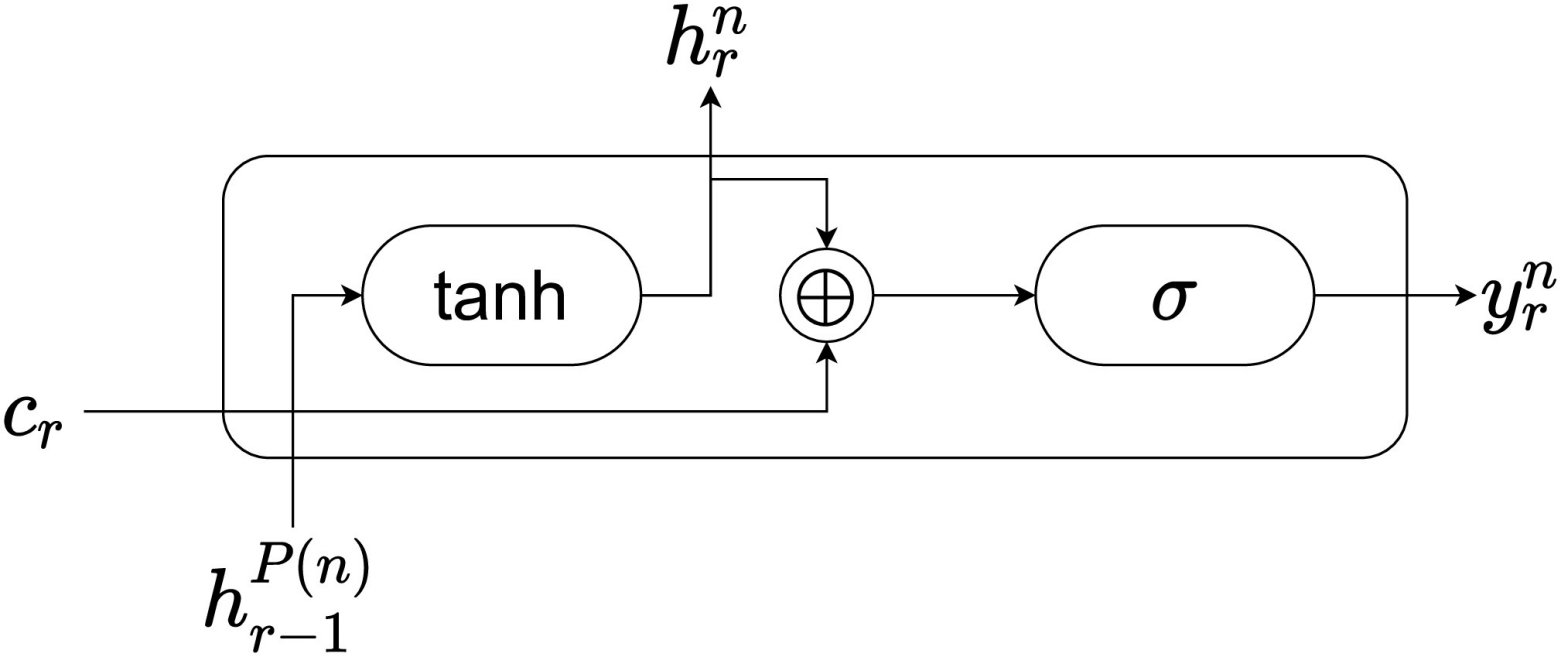
Unit of the decoder.

From the above, it is necessary for the decoder unit to be a model that receives input from the parent node and the context vector by attention, and outputs two outputs: one to the child nodes and the other to the entire model (predicted value). As mentioned earlier, the number of nodes will be very large, so we cannot introduce a very complicated model. The unit structure is shown in Fig 7.

The dimensions of the output to the intermediate layer and to the child nodes should be varied according to the complexity of the work to be done in that model. For example, if only two IPCs are predicted by the descendant nodes of a node, the dimension of the middle layer is considered to be small. On the other hand, if there are more than a hundred target IPCs, they should be embedded in a larger dimension to make them more expressive. Therefore, the dimensions of the middle layer are set according to the number of IPCs targeted by the descendant nodes. The output of the middle layer of the first node is given as a hyperparameter, and it is adjusted to be one-dimensional when there is only one target IPC. In the preliminary experiments, we compared two ways of changing the dimensions of the middle layer—linear and logarithmic—and adopted the logarithmic one. As a result, the number of IPCs owned by node *n* is *l*(*n*), the number of IPCs to be predicted by the model is *L*, and the feature dimension of the encoder is *D*.

$$\begin{array}{c} \text{dim}\;h_{r}^{n}=1+\left\lceil \left(D-1\right)\;{\text{log}}_{L}\;l\left(n\right)\right\rceil \end{array}$$

Based on the above, the forward propagation calculation of the entire decoder is expressed as follows, assuming that the output of the encoder is *h*_*enc*_ and *P*(*n*) is the parent node of node *n*.

$$c_{1}=\text{MHA}(q_{1},h_{enc},h_{enc})$$ (8)

$$h_{1}^{n}=\text{tanh}({W_{1}}_{1}^{n}c_{1}+{b_{1}}_{1}^{n})$$ (9)

$$y_{1}^{n}=\sigma ({W_{2}}_{1}^{n}h_{1}^{n}+{b_{2}}_{1}^{n})$$ (10)

$$q_{r}=V_{r-1}y_{r-1}+b_{r-1}\;\left(r\geq 2\right)$$ (11)

$$c_{r}=\text{MHA}(Q_{r},h_{enc},h_{enc})\;\left(r\geq 2\right)$$ (12)

$$h_{r}^{n}=\text{tanh}({W_{1}}_{r}^{n}h_{r-1}^{P(n)}+{b_{1}}_{r}^{n})\;\left(r\geq 2\right)$$ (13)

$$y_{r}^{n}=\sigma ({W_{2}}_{r}^{n}\left(h_{r}^{n}⊕c_{r}\right)+{b_{2}}_{r}^{n})\;\left(r\geq 2\right)$$ (14)

## 5 Experiment

To evaluate the effectiveness of the proposed method and its practicality, we experimented with the task of predicting IPC using real data against which existing methods were compared.

### 5.1 Experimental setup

We used real data registered as U.S. patents for the experiments. For the training data, it was assumed that the application was filed between 2010 and 2012 and registered by 2020. For the test data, we assumed that the applications were filed during the following year (2013) and registered by 2020. We obtained these data through PatentSQUARE(a fee-based patent data service). As a result, the total number of training data was 733,154, of which 10% were used as validation data. In contrast, the number of test data points was 261,622. The list of data counts is summarized in Table 1.

**Table 1:** Details of the number of data.

		SECTION							
	ALL	A	B	C	D	E	F	G	H
train	648861	106431	103568	74506	3926	17946	54594	240033	217001
valid	72096	11685	11517	8291	422	1973	6089	26674	24204
test	83032	15328	14661	9614	557	2695	8026	32757	29586

The preprocessing procedure is as follows. We use the Python NLTK library [18] to extract nouns and collect only the 512 nouns with the highest number of occurrences in each sentence that were used as inputs. If there were multiple occurrences of the 512th most frequent one, it was selected at random. The IPCs to be predicted were those that appeared in more than 100 cases in the training data (4087 labels in total). As a result, about three IPCs are granted per patent. Although some patent documents without any IPCs appeared in the training data, we did not exclude them because we thought they could not be excluded in practice. To avoid overfitting, dropout and layer normalize were used. For the hyperparameters of the model, the encoder dimensionality was 256, the depth was 4, the dropout rate was 0.2, and the regularization weight was 10^−5^. The first 10 epochs were trained by freezing the embedding layer using an Adam. After that, the freeze was removed, and all layers were trained using Adam for five epochs. The implementation was done using the Tensorflow module in Python.

### 5.2 Evaluation metrics

In previous studies, various evaluation metrics have been used owing to their different objectives. In [10], the evaluation metrics were precision, recall, and F-measure for all the prediction labels at all levels, because it is necessary to hit all the prediction labels equally. However, Patent BERT uses the prediction accuracy of the single label that it is most confident in predicting, which is not considered practical. Therefore, in this study, we provided a practical evaluation metric.

In predicting the IPC of a patent document, only the terminal labels are important. For example, even if the main group is correct but the subgroup is wrong, it is considered to be an erroneous assignment and meaningless. Therefore, we consider only whether the terminal subgroups are correct. Here, the question is how to evaluate the prediction accuracy of the terminal labels. This multi-label classification problem is considered a multiple-binary classification problem. Here, the percentage of specific IPC assigned in most cases is extremely small. Therefore, we evaluated the model using the metrics of the unbalanced label binary classification problem.

In binary classification problems, there are generally four patterns: the number predicted to be True and is True (True Positive; TP), the number predicted to be True and is False (False Positive; FP), the number predicted to be False and is True (False Negative; FN), and the number predicted to be False and is False (True Negative; TN).

actualTrueFalsepredictTrueFalseTPFPFNTN

Based on these, the precision, recall, and F-measure were obtained as follows, respectively.

$$\begin{array}{c} \text{Precision}=\frac{TP}{TP+FP} \end{array}$$

$$\begin{array}{c} \text{Recall}=\frac{TP}{TP+FN} \end{array}$$

$$\begin{array}{c} F-\text{Measure}=\frac{2\times \text{Precision}\times \text{Recall}}{\text{Precision}+\text{Recall}} \end{array}$$

These evaluation indices are affected by the threshold for the probability of granting a prediction. Therefore, we used the AUC as a method to evaluate only the model.

In addition, although the evaluation indices have been used to achieve full automation, we assume a situation in which patent examiners refer to them when granting IPCs and view the system as one that recommends those judged to have a high probability of being granted. For this purpose, recall Recall@*N* was introduced. This is to obtain recall when the top *N* of the probability of granting is predicted to be granted. This assumes that the patent examiner is recommended N IPCs that are expected to be granted, and the examiner selects from among them to grant. With this evaluation index, it is possible to evaluate how many correct IPCs are included in the N recommendations.

### 5.3 Results

First, a simple experiment was conducted to determine if it is appropriate to extract nouns. Specifically, because predicting all labels would be time-consuming, we limited the target labels to sections only and compared the metrics for parts of speech. Comparisons were made between verbs and adjectives, as well as words that obtained the highest number of occurrences regardless of part of speech. Experiments were conducted with two input word count patterns: 128 words and 512 words. The proposed encoder was used as the encoder, and a simple encoder consisting of only one matrix calculation and a sigmoid function was used as the decoder. The results are shown in Table 2.

**Table 2:** 8 label classification with each part of speech.

part of speech	number of words	Precision	Recall	F-measure	AUC
Noun	128	0.799	0.808	0.803	0.971
	512	0.805	0.805	0.805	0.972
Verb	128	0.692	0.693	0.692	0.937
	512	0.685	0.691	0.688	0.935
Adjective	128	0.731	0.725	9.727	0.949
	512	0.726	0.727	0.726	0.949
ALL	128	0.781	0.78	0.78	0.966
	512	0.812	0.810	0.811	0.973

We see that when 512 tokens are input, both nouns and whole words are more accurate. On the other hand, when the number of words is reduced to 128, the evaluations of nouns does not drop as much, whereas the evaluations of the model with all parts of speech input drops. If the number of input tokens is sufficient, it is slightly more accurate to input all parts of speech, but when processing a large number of predictive labels, it is better to reduce the input as much as possible to increase memory efficiency. Therefore, inputting nouns is the best way to process a large number of predictive labels. Here, adjectives are more accurate than verbs, which can be attributed to the fact that there are about 400,000 adjectives in the training data, while there are about 50,000 verbs. This result indicates that nouns contain more information for technical content classification than other parts of speech and are more effective in compressing information.

In the next experiment, we tested the metrics of the proposed model for each level of the hierarchy. The results are presented in Table 3. It can be seen that the prediction accuracy of the section is high, and the prediction accuracy decreases as we move down the hierarchy. In particular, the prediction accuracy of the main group and the subgroups decreased, but the reason may be that the decoding features were too small. It is important to identify the cause of this problem and to handle it to perform IPC prediction with high accuracy.

**Table 3:** Evaluation by level.

rank	Number of labels	Precision	Recall	F-measure	AUC
section	8	0.795	0.794	0.795	0.955
class	94	0.671	0.676	0.673	0.971
subclass	305	0.565	0.562	0.563	0.964
maingroup	1263	0.390	0.392	0.391	0.950
subgroup	4087	0.243	0.263	0.252	0.927

Next, to compare the prediction accuracy for each field, we calculated the prediction accuracy for each section. The results are shown in Table 4.

**Table 4:** Evaluation by section.

section	Average number of labeled patents	Precision	Recall	F-measure	AUC
A	324.8	0.263	0.319	0.288	0.935
B	231.9	0.236	0.151	0.184	0.883
C	248.7	0.260	0.220	0.238	0.922
D	122.3	0.218	0.148	0.177	0.884
E	178.0	0.170	0.138	0.152	0.913
F	212.7	0.195	0.226	0.210	0.918
G	521.3	0.281	0.277	0.279	0.928
H	408.0	0.232	0.240	0.236	0.928

This result shows that the accuracies of A and G are high, while the accuracies of B, D, and E are low. This result is generally higher for those with a larger number of data points per label. Therefore, there are many labels that have not reached a sufficient amount of data, and it is expected that more accurate predictions can be made if more data are available.

Next, we trained a model to predict all labels and compared it with existing methods. As a baseline, we compared with HARNN [10] and Patent BERT [7]. The model size of the HARNN was set to 256 dimensions along with the proposed model, and as in the proposed model, the first claim was used as the input. The word2vec model was used as the initial value for the embedding layer. The model size of the HARNN is 256 dimensions along with the proposed model, and the word2vec model is used as the initial value of the embedding layer, as in the proposed model. A 12-layer, 768-hidden, 12-heads model [15] was used as the BERT of Patent Bert.

**Fig 8:**
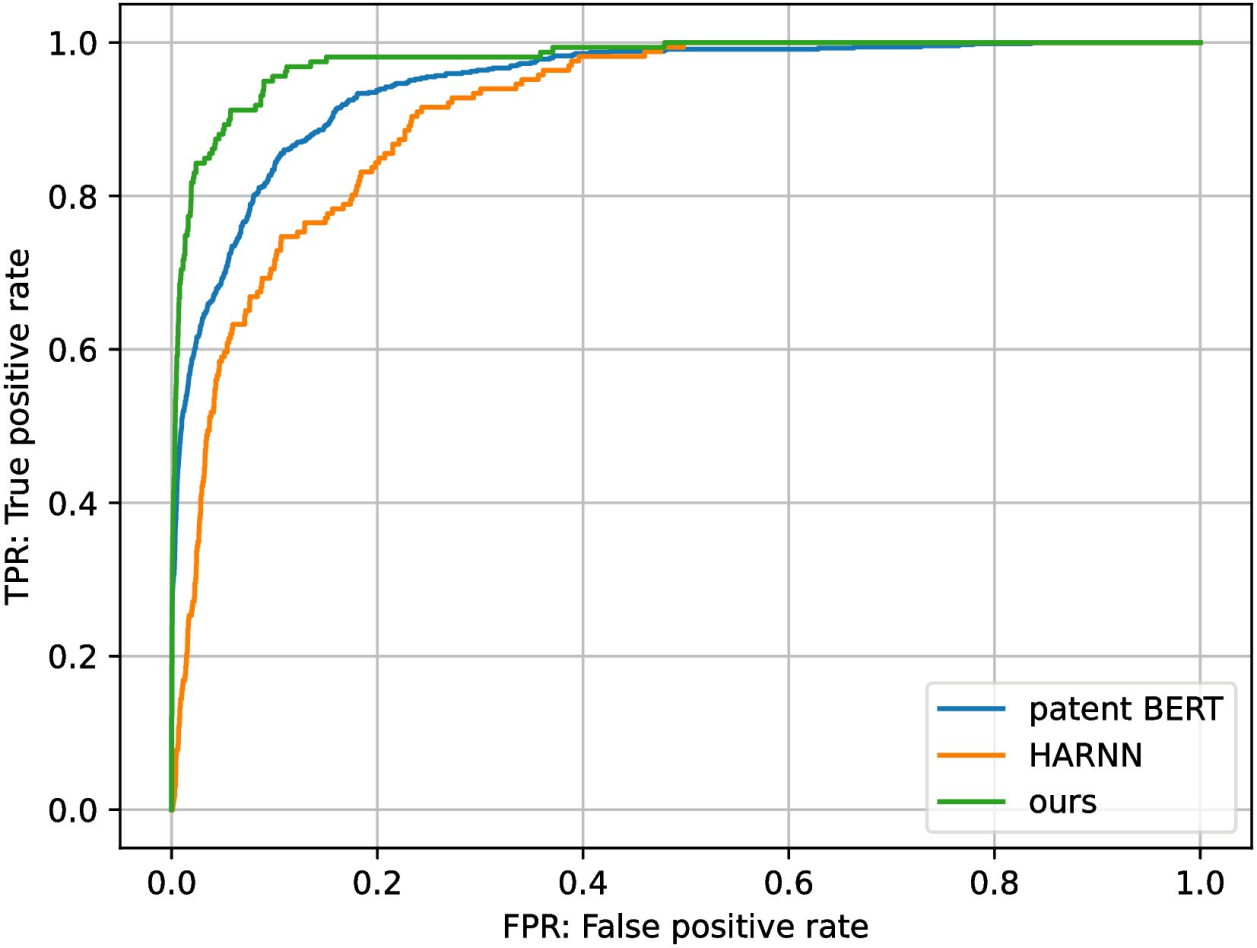
ROC-curve of subgroup classification.

The results of each evaluation index are listed in Table 5 and the ROC curve of this experiment is Fig 8. The results show that the proposed method significantly improves all evaluation metrics compared to the existing methods. Therefore, it can be said that the proposed method is more effective than the existing method patent BERT. In addition, when we compare patent BERT with HARNN, the AUC is not very different, but there is a large difference in F-measure, indicating that the effect of pretraining is significant. While the accuracy of the proposed model is relatively higher than that of the two existing methods, the F-measure is approximately 0.25 in absolute terms. This means that the proposed model is not yet at a practical level when it is used as a tool to fully automate the IPC assignment.

**Table 5:** Comparison with existing methods.

model	Precision	Recall	F-measure	AUC
ours	**0.243**	**0.263**	**0.252**	**0.925**
HARNN [10]	0.128	0.126	0.124	0.812
patent BERT [7]	0.194	0.194	0.195	0.806

**Fig 9:**
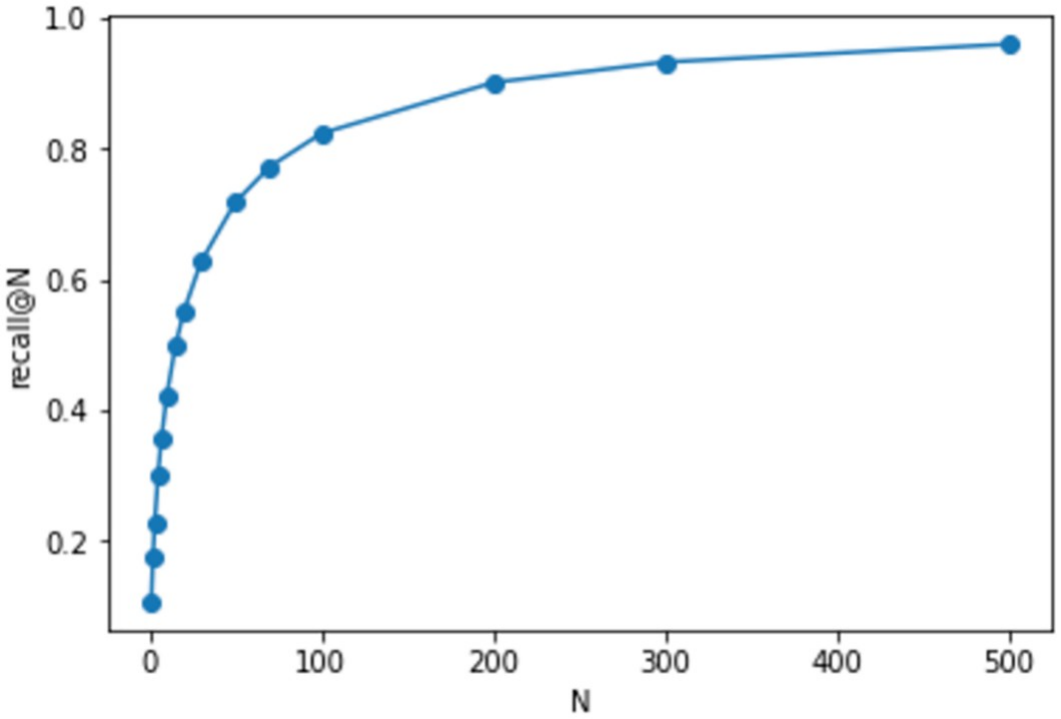
Result of recall@N.

Finally, to analyze the usefulness of the proposed model when used as a recommendation tool for patent examiners, Recall@N for the proposed method is shown in Fig 9. This shows that if we look at 30 labels, we can cover 70% of the labels, and if we look at 300 labels, we can cover 95% of the labels. Although it is impractical to look at 300 labels, it may be worthwhile to use this method to look at the top 30 labels to prevent IPC from being overlooked.

## 6 Conclusion and future work

In this paper, we propose a method for IPC assignment of patent documents. The first is to extract nouns and their percentages from all claims, and the second is a decoder to process locally specific information while taking global attention to handling the hierarchical structure of IPC. The second is a decoder that processes locally specific information while taking global attention to support the IPC hierarchy. We also used Recall@N to test the model when it was used to recommend labels that were likely to be assigned.

There are two issues that need to be addressed in the future. The first is to improve the feature extraction method. To reduce the amount of memory used in the model, we extracted nouns and their percentages as input, but this is a kind of compromise. It is true that nouns are the parts of speech that affect prediction more than other parts of speech, but information compression methods other than noun extraction should also be considered. There is also the possibility of further improving the accuracy of the feature extraction by utilizing other information such as the dependency relations of sentences and claims. Therefore, various feature extraction methods should be compared.

The second was to validate the prediction against a larger number of target labels. In the experiments conducted in this study, the prediction was performed for 4087 labels. However, there are approximately 70,000 IPCs in the real world, and there is a possibility of memory shortage and loss of accuracy if predictions are made on this scale. Therefore, it is necessary to verify whether learning is possible when the number of labels is increased, as well as the prediction accuracy. If it is not possible to learn, it may be necessary to divide the data into separate parts and predict them in some way.
